# Fluorolabeling of the PPTase-Related Chemical Tags: Comparative Study of Different Membrane Receptors and Different Fluorophores in the Labeling Reactions

**DOI:** 10.3389/fmolb.2020.00195

**Published:** 2020-08-07

**Authors:** Rosy Amodeo, Domenica Convertino, Mariantonietta Calvello, Lorenzo Ceccarelli, Fulvio Bonsignore, Cosetta Ravelli, Antonino Cattaneo, Claudia Martini, Stefano Luin, Stefania Mitola, Giovanni Signore, Laura Marchetti

**Affiliations:** ^1^NEST, Scuola Normale Superiore, Pisa, Italy; ^2^Center for Nanotechnology Innovation @NEST, Istituto Italiano di Tecnologia, Pisa, Italy; ^3^Bio@SNS, Scuola Normale Superiore, Pisa, Italy; ^4^Dipartimento di Farmacia, Università di Pisa, Pisa, Italy; ^5^Department of Molecular and Translational Medicine, University of Brescia, Brescia, Italy; ^6^CNR-NANO, Pisa, Italy; ^7^Fondazione Pisana per la Scienza Onlus, Pisa, Italy

**Keywords:** fluorophore, Single molecule labeling, PPTase labeling, Fluorescent Nerve Growth Factor, chemical tag, surface receptor

## Abstract

The set-up of an advanced imaging experiment requires a careful selection of suitable labeling strategies and fluorophores for the tagging of the molecules of interest. Here we provide an experimental workflow to allow evaluation of fluorolabeling performance of the chemical tags target of phosphopantetheinyl transferase enzymes (PPTases), once inserted in the sequence of different proteins of interest. First, S6 peptide tag was fused to three different single-pass transmembrane proteins (the tyrosine receptor kinases TrkA and VEGFR2 and the tumor necrosis factor receptor p75NTR), providing evidence that all of them can be conveniently albeit differently labeled. Moreover, we chose the S6-tagged TrkA construct to test eight different organic fluorophores for the PPTase labeling of membrane receptors in living cells. We systematically compared their non-specific internalization when added to a S6-tag negative cell culture, the percentage of S6-TrkA expressing cells effectively labeled and the relative mean fluorescence intensity, their photostability upon conjugation, and ratio of specific (cellular) versus background (glass-adhered) signal. This allowed to identify which fluorophores are actually recommended for these labeling reactions. Finally, we compared the PPTase labeling of a purified, YBBR-tagged Nerve Growth Factor with two differently charged organic dyes. We detected some batch-to-batch variability in the labeling yield, regardless of the fluorophore used. However, upon purification of the fluorescent species and incubation with living primary DRG neurons, no significant difference could be appreciated in both internalization and axonal transport of the labeled neurotrophins.

## Introduction

The choice of the right labeling strategies to monitor molecules of interest in living specimens represents a focal point when planning imaging measurements. In particular, the set-up of single molecule (SM) imaging experiments requires fluorophores with peculiar photophysical properties as high photostability, brightness, ability to photoactivate, or to photoswitch (Fernández-Suárez and Ting, 2008). Accordingly, several novel classes of organic dyes have been developed in the last years (Zheng et al., 2014; Grimm et al., 2016; Li and Vaughan, 2018), along with the parallel development of chemical tags (Jing and Cornish, 2003; Keppler et al., 2003; Gautier et al., 2008), or alternative strategies (Teng et al., 2016) to achieve labeling of membrane and intracellular proteins with the desired organic dye. Despite the recognized advantages offered by these methodological approaches, there are still challenges to overcome. The most notable is probably non-specific interaction of the dyes with lipid bilayers, which can cause both alteration of labeling specificity and generation of false signals due to the fluorophore attachment to the plasma membrane (Hughes et al., 2014). Therefore, the choice of the correct fluorophores for SM imaging experiments typically benefits from, and often requires, a preliminary step of optimization. For example, considerations about the physiological expression level of the protein to be labeled, and to what extent its conjugation to a peculiar fluorophore can produce a signal higher than the background must be taken into account. Similarly, fluorophores need to be chosen depending on the experimental set up available in the microscope. An interesting report, using a SNAP-tag fusion construct of the EGF receptor, showed that not all dyes are suitable for the same purposes, because of their different photostability and specificity in the conjugates required for SNAP-tag labeling (Bosch et al., 2014). Another group screened nine different fluorophores to understand the best fluorophores-lipid combination to study changes in conformation of proteins embedded in lipid vesicles with SM resolution (Zhang et al., 2017).

In the last years, our group has exploited the acyl and peptidyl carrier protein (ACP and PCP) derived chemical tags applied to the labeling of neurotrophins and their receptors, respectively, acting on the purified protein and in a living cell context (Callegari et al., 2012; Gobbo et al., 2018; Marchetti et al., 2019; Amodeo et al., 2020). A great advantage of this labeling technology is that these short peptides, once inserted in the protein of interest, have low steric footprint on its structure and function. Tag recognition by phosphopantetheinyl transferase enzymes (PPTases) allows its easy, covalent labeling with 1:1 stoichiometry to any probe that can be derivatized with a Coenzyme A (CoA) substrate (Johnsson et al., 2005). Indeed, PPTases catalyze the transfer of the functionalized CoA PP arm to a serine residue of the tag. Using this strategy, we achieved the first fluorophore labeling with definite stoichiometry of Nerve Growth Factor (NGF; De Nadai et al., 2016; Gobbo et al., 2018). Here, we report a rational optimization process that guided our experimental choices and may serve as a general guideline for chemical tag labeling approaches. Our results focus on three different aspects related to chemical tag labeling. First, we asked what is the performance of the same chemical tag when this is fused to structurally or functionally different single-pass transmembrane receptors; accordingly, we compared the labeling reactions of a S6 tag fused to the N-terminal sequences of TrkA, P75NTR, and VEGFR2 receptors. Second, we analyzed how the labeling of a single membrane receptor is influenced by the use of different CoA-fluorophore conjugates in the labeling reaction, by testing eight different CoA-fluorophore substrates for labeling of S6-TrkA receptor. Finally, we investigated if the fluorophore charge influences internalization and trafficking of a labeled species inside a living cell. We addressed the last question by fluorolabeling NGF-YBBR protein with two differently charged fluorophores and evaluating fluoNGF internalization and trafficking inside living neurons.

## Materials and Methods

### Cell Surface Labeling of Different Receptors

SHSY5Y cells expressing the TrkA, P75NTR, or VEGFR2 constructs were starved for 2 h in a non-supplemented medium containing 0.5% BSA (starvation medium), and thereafter labeled for 30 min at 37°C with a mix containing 2 μM Sfp synthase, 10 mM MgCl_2_, and 10 μM CoA-biotin; alternatively, with a mix containing 2 μM Sfp synthase, 10 mM MgCl_2_ and 500 nM CoA-Alexa647 in starvation medium. The former reaction was washed two times in PBS, and cells further incubated for 2 min at room temperature with 10 nM S-Qdot (Qdot^®^ 655 streptavidin conjugate; Invitrogen) in borate buffer pH 8.3, 0.5% BSA and 215 mM sucrose (Callegari et al., 2012). Cells were washed five to eight times with PBS, and then imaged in 20 mM HEPES, 6.6 mM D-glucose, 2 mM L-glutamine, 1 mM sodium pyruvate and 0.5% B-27 supplement in MEM medium at the Total Internal Reflection Fluorescence (TIRF) microscope. Quantification of the relative abundance of cell surface receptors was performed by calculating the mean intensity of Qdot and Alexa647 positive cells in the different acquired fields after background subtraction. Results are reported as mean ± s.e.m.

### Labeling of S6-TrkA Single Molecules With Different CoA-Fluorophore Substrates

The synthesis of CoA-Alexa488, CoA-Abberior488, CoA-Atto488, CoA-Atto550, CoA-Alexa568, CoA-Atto633, CoA-Alexa647, CoA-Abberior 635P substrates was performed as described previously (Marchetti et al., 2013, 2014; Di Matteo et al., 2017). 48–64 h after transduction with S6-TrkA construct, SHSY5Y cells were seeded into Willco-dish^®^ glass-bottom dishes and supplemented with 1 μg/ml doxycycline for at least 24 h. Cells were then starved for 2 h, incubated for 30 min at 37°C with a mix containing 2 μM Sfp synthase, 10 mM MgCl_2_, and in turn 500 nM of the aforementioned coenzyme A-fluorophores. In parallel, non-transduced SHSY5Y cells were incubated for 30 min at 37°C in DMEM-F12 containing 0.5% BSA, 10 mM MgCl_2_, and 500 nM of every CoA-fluorophore, to test non-specific interaction of the fluorophores with the plasma membranes and subsequent cellular signal. In any case, cells were washed five times with PBS and imaged at the TIRF microscope. For each fluorophore we quantified: (i) the labeled cell fraction, i.e., the average percentage of labeled cells in 4–7 acquired fields for each fluorophore tested; in each field, only cells displaying a sizeable (>10) amount of moving spots at the cell surface were considered labeled; (ii) the mean normalized intensity of labeled cells, i.e., the mean value of the background-subtracted average intensity of labeled cells as in (i), divided for the brightness of each fluorophore; note that this normalization only allows for comparative evaluation within the same fluorophore category (green, red, far-red); (iii) fluorophore photostability, by averaging the value [1-(I_post_/I_pre_)] obtained for 13–30 different cells in different selected fields (I_post_ and I_pre_ are the mean intensity of each cell in the last and first frame of a 500-frame time series of the selected field, respectively); note that in this case, photobleaching was comparatively evaluated within the same fluorophore category (green, red, and far-red) subjected to the same bleaching procedure. Laser power and other experimental parameters were kept constant within each fluorophore class; (iv) the signal-to-background ratio (S/Bckg), i.e., the ratio between the previous I_pre_ values and the average background value obtained from 3 selected glass areas around each labeled cell; and (v) non-specific interaction with cells, by averaging the fluorescence intensity of about 20 non-transduced cells per field, in 5 fields per CoA-fluorophore, and dividing this value for the same one obtained for cells not incubated with any CoA-fluorophore, imaged at the same microscope set-up. Effectively, we obtained the field average intensity value by ImageJ software, measuring the mean intensity of the whole field after background subtraction and application of a threshold to remove pixels outside the cells. In these conditions, we found that background signal was the same for cells incubated with or without CoA-fluorophores. This result suggests that most of aspecific adhesion to the glass surface is triggered by PPTase addition to the cell medium. Results are reported as mean ± s.e.m., after normalization to the signal of cells not incubated with CoA-fluorophores, imaged with the relative microscope set up. All shown images were subjected to background subtraction and linear modification of brightness and contrast.

### fluoNGF Axonal Transport in DRG Neurons

To study fluoNGF axonal transport, the axon compartment of DIV4-5 DRG neurons was starved for 1 h from NGF and then supplemented with 2 nM fluoNGF. Live cell imaging was performed in the channel compartment after 50 min from fluoNGF administration at the TIRF microscope. FluoNGF positive vesicles were tracked over time with homemade scripts in MatLab (The MathWorks) implementing the functions used by Raghuveer Parthasarathy (Parthasarathy, 2012; De Nadai et al., 2016). For each experiment we collected at least 1000 trajectories. Trajectories shorter than 30 frames were discarded, the others were considered “anterograde” or “retrograde” depending on the direction of the motion, respectively, from the axons to the soma or vice versa. Using a mobile window of 5 frames to calculate the average vesicle velocity, we considered positive the retrograde transport and negative the anterograde transport applying a threshold of 0.5 μm/s (−0.5 μm/s) for retrograde (anterograde) motion. Vesicles slower than the threshold were only used, together with the moving ones, to calculate the total amount of internalized vesicles.

### TIRF (Total Internal Reflection Fluorescence) Microscopy

Cells were imaged at 37°C, 5% CO_2_ using an inverted epi-fluorescence microscope (Leica AF6000) equipped with Leica TIRF-AM module, incubator chamber, electron multiplying charge-coupled-device camera (ImagEM C9100-13, Hamamatsu), and 100 × oil immersion objective (NA 1.47), allowing the acquisition of fields of 512 × 512 pixels (116.80 × 116.80 μm) typically comprising several cells, or two channels of the compartmentalized DRG culture. TIRF images of Qdot655 labeled receptors were acquired using a 488 nm laser line with a penetration depth set at 90 nm and FF01-655/15 Semrock emission filter. Abberior635, Alexa647, and Atto633 were imaged using the 635 nm laser line with a 110 nm penetration depth for excitation and a Cy5 Leica1152303 emission filter. Atto550 and Alexa568 were imaged using the 561 nm laser line with a 350 nm penetration depth and a RFP Leica513894 filter cube. Abberior488, Alexa488, and Atto488 were imaged using the 488 nm laser line with 90 nm penetration depth for excitation, a 482–510 excitation filter and a BP 525/20 Leica emission filter. Laser power, gain and EM gain values were kept constant within different groups to allow quantitative comparisons; exposure times were 45 ms for Abberior488, Alexa488, and Atto488; 60 ms for Atto550 and Alexa568; and 40 ms for Abberior635, Alexa647, and Atto633. FluoNGF conjugated to Alexa488 and Abberior488 was excited with a 488 nm laser, the TIRF module set in epifluorescence mode, and a constant exposure time of 100 ms. Up to 1000 frames for each time-serie were acquired to track fluoNGF vesicles in DRG axons.

### Data Analysis

Statistical analysis was performed with GraphPad Prism 6 software. Parametric tests used were *T*-test for the comparison of two groups, and one-way ANOVA, with Bonferroni’s comparison of means, when analyzing more than two groups. Statistical evaluation of NGF vesicles number was performed with non-parametric Mann-Whitney test. Significance was set at α = 0.05. Data are represented as mean ± s.e.m., unless otherwise stated. The *n* and *P* values for all experiments are reported in the relative figure captions.

## Results and Discussion

### Comparative Qdot- and Fluorophore- Labeling of Different S6-Tagged Membrane Receptors

**FIGURE 1:**
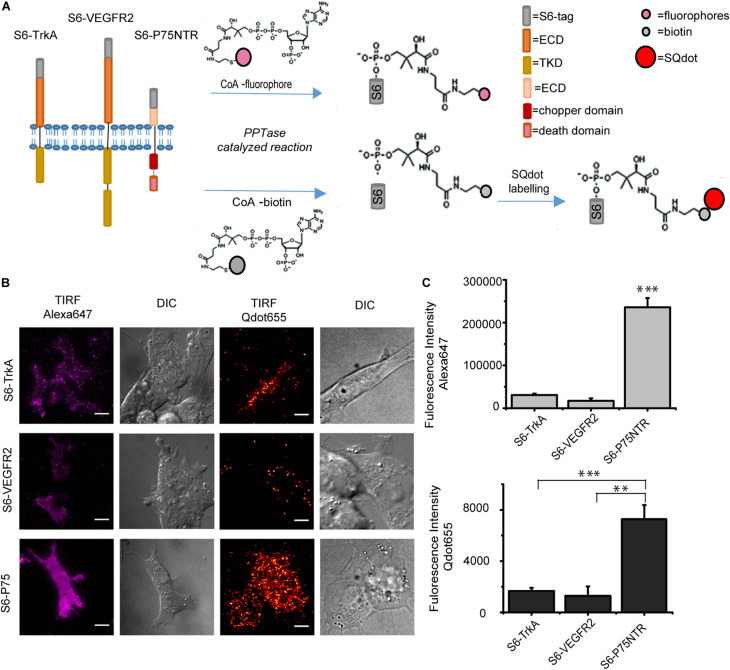
**(A)** Scheme of the PPTase-based labeling of S6-tagged TrkA, VEGFR2, and P75NTR receptors by CoA-conjugates. S6 tag exhibits the hydroxyl group of a serine residue recognized by the PPtase. Top: conjugation of the PPant arm of CoA-fluorophore conjugate at serine –OH group; bottom: conjugation of the PPant arm of CoA-biotin at serine –OH group; biotinylation precedes the labeling step with streptavidin-coated Qdots (S-Qdot). **(B)** TIRF and corresponding DIC microscopy images of SHSY5Y cells expressing S6-TrkA (top), S6-VEGFR2 (middle), and S6-P75NTR (bottom), after fluorolabeling by PPTases. On the left, S6-tagged receptors labeled with 500 nM CoA-Alexa647; on the right, labeling with 10 nM S-Qdot; and scale bar: 10 μm. **(C)** Quantification of labeling efficiency of experiments shown in panel **(B)**. Top: plot of mean fluorescence intensity (±s.e.m.) of Alexa647-labeled S6-TrkA (*n* = 49 cells), S6-VEGFR2 (*n* = 7 cells), and S6-P75NTR (*n* = 80 cells). Bottom: plot of mean fluorescence intensity (±s.e.m.) of S-Qdot-labeled S6-TrkA (*n* = 17 cells), S6-VEGFR2 (*n* = 6 cells), and S6-P75NTR (*n* = 18 cells). ^∗∗∗^*P* < 0.001, ^∗∗^*P* < 0.01 following one-way ANOVA test, with Bonferroni’s comparison of means.

TrkA, VEGFR2, and P75NTR are single-pass transmembrane receptors differing both at the structural and functional levels. TrkA and VEGFR2 belong to the family of receptor tyrosine kinases (RTKs; Hubbard and Miller, 2008), while P75NTR belongs to the family of tumor necrosis factor receptors (TNFRs; Vilar, 2017; Figure 1A). Also, TrkA and p75NTR are master regulators of the neurotrophic responses in neuronal cells (Marchetti et al., 2015), while VEGFR2 is a pro-angiogenic receptor of endothelial cells (Damioli et al., 2017). From the chemical labeling perspective, their extracellular domains (ECDs) have different lengths, with p75NTR displaying the shortest and VEGFR2 the longest distance from cell surface. Cloning of the S6 tag sequence at the ECD of the three of them previously allowed for their labeling and imaging with SM resolution on the cell surface in living cells (Marchetti et al., 2019; Amodeo et al., 2020). Here, we compared how the same labeling reactions perform on these three moieties, considering also their different exposure from the cell membrane, which may be relevant for enzymatic recognition of the tag. S6-tagged TrkA, P75NTR, and VEGFR2 were expressed in SHSY5Y cells, and S6 tag was either biotinylated and then coupled to streptavidin-coated Qdots, or directly conjugated to Alexa647 (Gobbo et al., 2018). In both cases, labeled cells were quantitatively analyzed by TIRF microscopy (Figure 1B). We found a significant, ∼7- to 10- fold higher fluorescence signal for both labeling reactions on p75NTR compared to TrkA and VEGFR2 expressing cells. However, for the VEGFR2 case only a low number of cells were found to be effectively labeled (Figure 1C). These differences were confirmed by parallel experiments in which the S6 tag was biotinylated and then pulled-down with streptavidin-coated beads. As shown in Supplementary Figure 1, also in this case the biotinylated p75NTR band was dramatically higher than the RTKs ones, which in the same experimental conditions were barely visible only for the TrkA case. Overall, these results can be explained by two possible reasons: (i) a lower labeling efficiency of the S6 tag fused to RTKs compared to p75NTR; (ii) a lower surface abundance of RTKs (TrkA and VEGFR2) with respect to TNFRs (p75NTR). However, the presence of unlabeled species was never high enough to hamper detection of TrkA dimerization using this labeling approach (Amodeo et al., 2020), so that we believe the second reason to be the most likely. This in turn may depend on the different expression levels displayed by the P75NTR and the RTKs constructs, as witnessed by the lower number of labeled cells found for the latter (Figure 1C). Furthermore, we do not exclude that there may be also a relevant difference between the relative localization of the receptors, caused, e.g., by the fact that a considerable amount of membrane RTKs might reside in early/recycling endosomes proximal to the membrane bilayer, which are less available for the PPTase labeling reaction (Jopling et al., 2011). In any case, further experiments will be required, in which the S6 signal can be adequately normalized to an equal tag/antibody system in the three cases, to assess potential differences in S6 labeling yield for the three receptors.

To conclude, the previous data indicate that labeling by PPTase enzymes on cell surface receptors is not limited by the length of the ECD, provided that S6 tag is fused at its distal portion from the membrane; indeed, p75NTR ECD is the shortest one used in our experiments (Figure 1A), but is also the one displaying the highest number of labeled moieties. Surely, we have a lower limit of 230 amino acids on the ECD length and do not take possible ECD curvatures into account; however, successful S6 labeling previously reported for constructs of the Atypical Receptor CCRL2 suggests that this limit can be further scaled down (Mazzotti et al., 2017).

### Comparative Study of S6-TrkA Labeling by Different CoA-Fluorophore Substrates in the PPTase Reaction

**FIGURE 2:**
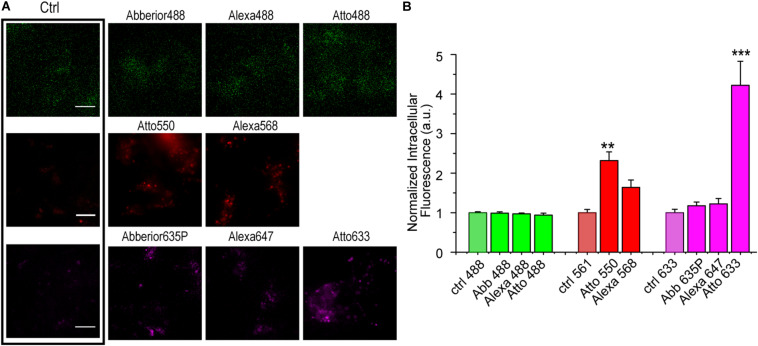
**(A)** Representative epifluorescence images of SHSY-5Y cells not transduced with S6-TrkA construct, incubated with 500 nM of each CoA-fluorophore for 30 min at 37°C. On the left, the box highlights the intensity images derived for cells not incubated with any CoA-dye and acquired at the same microscope set-up. Scale bar: 10 μm. **(B)** Quantification of intracellular fluorescence intensity (mean ± s.e.m.) of non-transduced SHSY5Y cells incubated with eight different CoA-fluorophores, normalized for cell autofluorescence intensity (ctrl) in the same emission channel (*n* = 5 fields, comprising at least 20 cells for each sample). ***P < 0.001, **P < 0.01 following one-way ANOVA test, with Bonferroni’s comparison of means.

To understand the effect of a fluorophore dye on S6 tag labeling efficiency, we screened the labeling performance of S6-tagged TrkA in living cells by eight different CoA-fluorophores, whose main photophysical features appear in Supplementary Table 1. SHSY5Y cells were either left untreated or transduced with a lentiviral vector carrying S6-TrkA, whose expression was induced by doxycycline addition to the cell medium (Gobbo et al., 2018). We first investigated the level of non-specific interaction of the different CoA-fluorophore substrates with cells not expressing S6-TrkA; cells were incubated with 500 nM of each fluorophore for the same time required for SfpS labeling and were imaged by TIRF microscopy (Figure 2A). Results, shown as mean intracellular fluorescence intensity normalized to the relative cell autofluorescence intensity in the same emission window, show that all green fluorophores (CoA-Abberior488, CoA-Alexa488, and CoA-Atto488) and two of the far-red fluorophores tested (CoA-Abberior635STAR-P, CoA-Alexa647) show negligible levels of non-specific internalization (Figure 2B). Conversely, we detected appreciable levels of intracellular fluorescence upon incubation with CoA-Atto550 and CoA-Alexa568, and a ≈ 4-fold increase for CoA-Atto633 (Figure 2B). We argue that, for the Atto550 and Atto633 cases, this unspecific signal is a direct consequence of the unspecific interaction of the fluorophore *per se* with cell membranes, as reported by other studies (Bosch et al., 2014; Hughes et al., 2014). This in turn may depend on the higher hydrophobicity in general displayed by Atto dyes when compared, e.g., to Alexa dyes [Supplementary Table 1 and (Patra et al., 2020)]. Furthermore, these two fluorophores bring a net positive charge in physiological conditions (Supplementary Table 1) which could also promote adhesion to cell membrane. As for CoA-Alexa568, given its low interaction factor with membranes (Hughes et al., 2014), and hydrophilic nature (Supplementary Table 1), we hypothesize that other mechanisms depending on Alexa568 conjugation to CoA may account for the observed unspecific intracellular signal.

**FIGURE 3:**
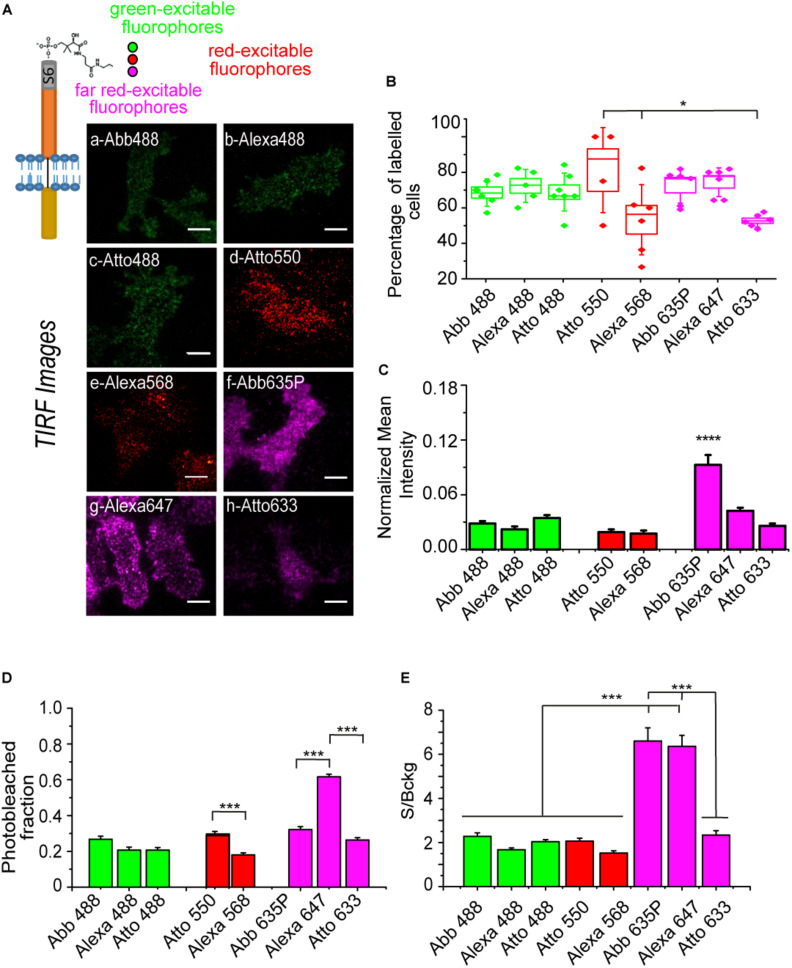
**(A)** Top-left: scheme of S6-TrkA receptor labeled with different CoA-fluorophores (with excitation in the green, red, far red range of the UV-visible spectrum). Right: representative background-subtracted TIRFm images of SHSY5Y cells expressing S6-TrkA and labeled with eight different fluorophores; scale bar: 10 μm. **(B)** Labeled cell fraction for each analyzed field, obtained from the % of the labeled cells present in 4–7 acquired fields; the box middle line corresponds to the average value, the limits to the s.e.m., and the whiskers to the st.dev. *P < 0.05 following one-way ANOVA test, with Bonferroni’s comparison of means (*n* = 6 fields for Abberior488, Alexa568, Abberior635P, Alexa647, and Atto633; *n* = 5 fields for Alexa488; *n* = 7 fields for Atto488; *n* = 4 fields for Atto550; and total analyzed cells in the fields ranged from 15 to 133). **(C)** Quantification of the mean fluorescence intensity (± s.e.m.) of S6-TrkA labeled cells for each fluorophore, normalized for the relative brightness value. ****P < 0.0001 following one-way ANOVA test, with Bonferroni’s comparison of means within each separate fluorophore class (*n* = 20, 14, 28 cells for Abberior488, Alexa488, and Atto488; *n* = 11, 21 cells for Atto550, Alexa568; *n* = 43, 56, 17 cells for Abberior635P, Alexa647, and Atto633). **(D)** Photobleached fraction (mean ± s.e.m.) for the different fluorofores, calculated from cells out of 6 different fields for each fluorophore. ***P < 0.001, *P < 0.05 following one-way ANOVA test, with Bonferroni’s comparison of means within each separate fluorophore class (*n* = 24, 20, 28 cells for Abberior488, Alexa488, and Atto488; n = 13, 22 cells for Atto 550, Alexa568; *n* = 30, 25, 23 cells for Abberior635P, Alexa647, and Atto633). **(E)** Histogram of the signal to background (S/Bckg) ratio (mean ± s.e.m.) evaluated for the same cells as in panel **(D)** for each fluorophore. ***P < 0.001, **P < 0.01 following one-way ANOVA test, with Bonferroni’s comparison of means.

We next performed the full labeling reaction on S6-TrkA transduced cells by using the same 8 fluorophores in the labeling mix (Figure 3). TIRF microscopy of the labeled cells allowed us to successfully identify labeled cells for each reaction, but with some differences, especially in cell fluorescence intensity with respect to the background (Figure 3A). Accordingly, we analyzed a series of parameters to compare more quantitatively the eight labeling reactions. We first evaluated the percentage of labeled versus total cells in the analyzed fields (Figure 3B). We observed high labeling percentages for all CoA-fluorophores tested, mostly ranging from ≈60 to 80%. However, for Alexa568, Atto550, and Atto633, we found that the number of labeled cells were slightly lower or displayed higher variability, when compared to the other samples; this could be due to the non-specific internalization of the CoA-fluorophore (Figure 2B), competing or interfering with the labeling performance. We also measured the mean intensity of the labeled cells (Supplementary Figure 2A). As this represents the convolution of both the number of receptors effectively labeled and the innate ability of the fluorophore to emit fluorescence, we normalized this signal for the brightness of each dye [i.e., the product of quantum yield and extinction coefficient reported in Supplementary Table 1 (Miyawaki et al., 2003)]. Results, reported in Figure 3C, show that while the considered green- and red- excitable fluorophores gave similar labeling yields within their class, this was different for the far-red ones, where labeling by the Abberior 635P dye displayed a significantly higher yield than by the Alexa 647 and Atto 633 dyes. The worse performance of Atto 633 could be caused by its aspecific internalization in cells (Figure 2B), which decreases the availability of substrates for the PPTase reaction. On the other hand, the worse performance of Alexa 647 is not obvious to explain based on its photophysical properties (Supplementary Table 1); it is possible that having a logD value close to zero with a negative charge (Supplementary Table 1) plays a role in increasing labeling efficiency for Abberior 635P.

Furthermore, we determined the photostability of each analyzed fluorophore when conjugated to S6-TrkA, by measuring the photobleached fraction after a 500-frame series for each fluorophore (Figure 3D): we concluded that, in our experimental conditions, all green-excitable fluorophores display similar bleaching. In the red region, performance of Atto550 is by far superior to that of Alexa568. Alexa647 shows significantly more extensive degradation than the two other far-red excitable fluorophores. Finally, we evaluated the labeling signal to background ratio (S/Bckg) for each fluorophore (Figure 3E): this parameter was empirically defined as the ratio of cell specific signal versus signal stemming from fluorophores not-specifically adhered to the glass, the latter being an unavoidable event occurring during PPTase labeling reactions. In these conditions, we found that only Abberior635P, and Alexa647 display S/Bckg significantly higher than the others.

Overall, these data indicate that Abberior635P is the best candidate for experiments for SMI of S6-TrkA receptor, explaining the choice of this fluorophore in previous experiments (Amodeo et al., 2020). Indeed, this fluorophore ensures optimal labeling efficiency and signal to background ratio while having relatively low photobleaching percentage and non-specific cell internalization. We do not exclude that, for labeling of other receptors displaying considerably higher membrane densities, as is the case of p75NTR (Figure 1), other fluorophores may also be suitable. This is supported by the analysis of the labeling, by the same eight dyes, of A1-P75NTR construct (Marchetti et al., 2014) expressed in SHSY5Y cells (Supplementary Figure 2B) for which we detected less evident differences than for the TrkA case (Figure 3C). Thus, we suggest that this analysis should be performed in a case-by-case manner to optimize labeling procedures according to the specific surface exposure displayed by each receptor type.

### Dependence of Internalization and Axonal Transport of NGF-YBBR on the Fluorophore Used for Its Fluorolabeling *in vitro*

**FIGURE 4:**
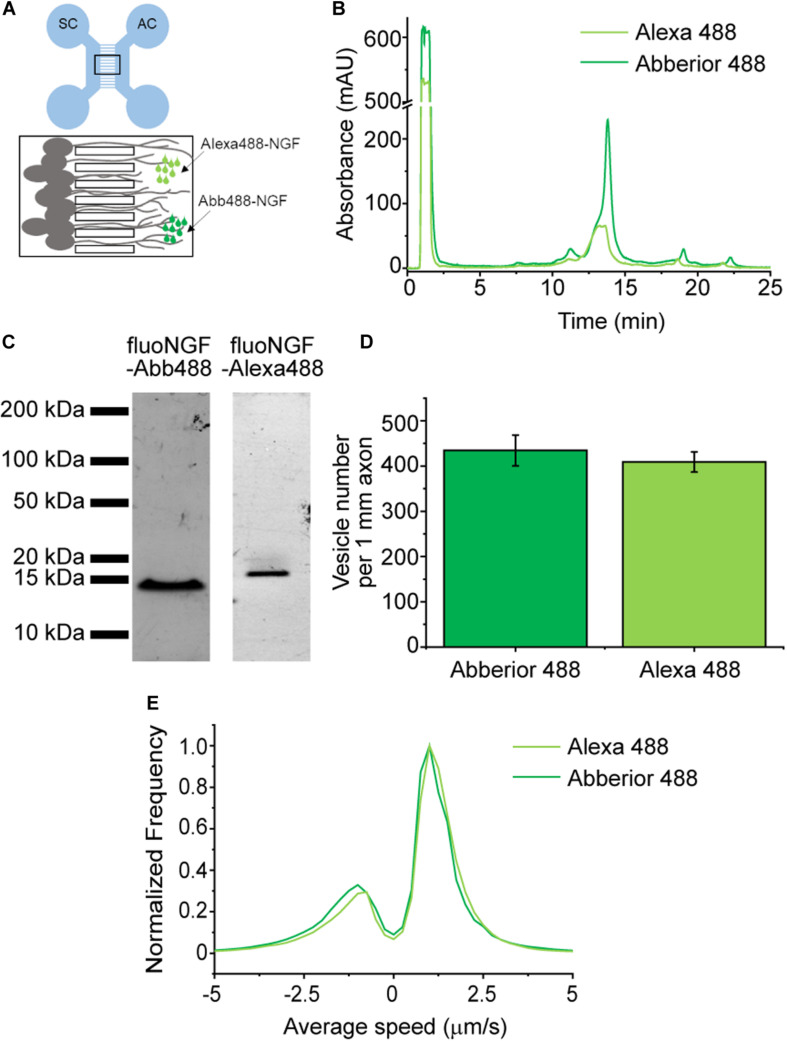
**(A)** Scheme of the microfluidic device, with the soma compartment (SC) connected to the axon compartment (AC) by the microgrooves (black box zoomed on the bottom). DRG neurons seeded in the SC extend their axons in the microgrooves reaching the AC where fluoNGF is administered. Image acquisition is performed in the microgrooves. **(B)** Superimposed HPLC chromatographic profiles of absorbance at 280 nm versus elution time of fluoNGF-Alexa488 (light green) and fluoNGF-Abberior488 (dark green) reactions. **(C)** SDS-PAGE analysis showing equal volumes of the eluted fractions, corresponding to ≈100 ng purified fluoNGF-Abberior488 (left) and ≈30 ng fluoNGF-Alexa488 (right), with the characteristic fluoNGF band at 14 kDa. Detection is performed by collecting fluorescence emitted at 510 nm. **(D)** Histogram of the mean ± s.e.m. of the number of vesicles per 1 mm of axons after fluoNGF-Alexa488 (*n* = 7674 vesicles) or fluoNGF-Abberior488 (*n* = 4089 vesicles) administration at the AC of DRG neurons. Data are not significantly different according to a Mann-Whitney test, with α = 0.05. The number of compartmentalized DRG cultures was 4 for NGF-Alexa488 and 2 for NGF-Abb488 with a total number of acquired vesicles of 7674 and 4089, respectively. **(E)** Normalized average speed distribution of moving vesicles (*n* = 1230 for fluoNGF-Alexa488 and 497 for fluoNGF-Abb488) referred to the same experiments as in panel **(D)**.

We analyzed the labeling performance of YBBR-tagged NGF by two different green excitable fluorophores (Supplementary Table 2), in order to understand whether both the labeling reaction and fluoNGF internalization in neurons are influenced by the fluorophore (Figure 4A). Indeed, NGF is a smaller moiety than receptor ECDs, and its labeling may affect protein folding and hence biological function. We thus produced in parallel two different preparations of fluoNGF using either CoA-Abberior488 and CoA-Alexa488 as enzyme substrates (Di Matteo et al., 2017). The two reactions were purified by cationic ion-exchange HPLC: the chromatograms in Figure 4B show that both species were recovered in sufficient amounts, with elution times of, respectively, 13.3 min and 13.8 min for NGF-Alexa488 and NGF-Abberior488. Retention times agree with the different charge of the two fluorophores (-3 and -2, respectively; see Supplementary Table 1), and with the different impact on overall protein charge. Also, the concentration and recovery yields were slightly higher for NGF-Abberior488 than for NGF-Alexa488, as confirmed by SDS-PAGE analysis of the eluted fractions (Figure 4C) and by quantification of fluoNGF concentration by spectrofluorimetry (De Nadai et al., 2016); however, this difference was not statistically significant and might also be due to some batch-to-batch variability in the labeling efficiency rather than to a different labeling performance of the two fluorophores (Supplementary Table 2).

The two purified fluoNGF forms were added to the axon compartment of compartmentalized chambers where DRG from neonatal mice were previously seeded (De Nadai et al., 2016; Convertino et al., 2020; Figure 4A). Fluorescence imaging of the retrograde and anterograde NGF vesicle flux in the channel compartment (black frame in Figure 4A), revealed that the total amount of internalized vesicles per 1 mm axon does not significantly differ for Alexa488 and Abberior488 conjugations (Figure 4D). Thus, the two different fluorophores, and the different resulting fluoNGF charge after their conjugation (Supplementary Table 2), do not affect the internalization process. We also quantified the distribution of speed of anterogradely and retrogradely moving fluoNGF vesicles, which are represented by positive and negative velocities, respectively (Figure 4E). In agreement with our previous observations (Gobbo et al., 2018), the number of vesicles transported retrogradely was significantly higher than those transported in the opposite direction. Importantly, both fluoNGF preparations showed a similar speed distribution, meaning that also the axonal transport properties are not affected by the chosen fluorophore.

## Conclusion

We propose here a systematic approach that may serve as a guideline for the chemical biology community to allow a robust, quantitative evaluation of fluorolabeling efficiency and behavior of chemical tags in high sensitivity microscopy setups. Indeed, our experimental workflow can be easily applied to virtually any protein/fluorophore combination. Here, we apply it to the case of chemical tags target of PPTase conjugations (Yin et al., 2005). Together with other works by our group and others (De Nadai et al., 2016; Di Matteo et al., 2017; Gobbo et al., 2018; Marchetti et al., 2019; Amodeo et al., 2020), we provide evidence for a good labeling performance of these tags both on purified proteins (Figure 4) and on membrane receptors of living cells (Figures 1–3). In the former case, we established, by means of experiments in living neurons, that the identity of the fluorophore does not affect biological function, provided that purification *in vitro* of the labeled protein is achieved. As for membrane receptor labeling, we observed a strict relationship between physico-chemical properties of the fluorophore and suitability of the labeling strategy for high-end microscopy techniques such as TIRF, with particular respect to signal-to-noise ratio and aspecific adsorption to the cell membranes or even to the glass surface. The latter can be limited by extensive wash of the labeled cells prior to imaging, however, some adsorbed fluorophore dots are always visible around labeled cells (Figure 1B and Supplementary Figure 3A). This adsorbed fluorophore pool may constitute the cause for the specificity limitations displayed by this method in recent protocols involving cell detachment by scraping after labeling (Stüber and Plückthun, 2019). However, for applications in which cells are directly imaged after the labeling reaction, the use of this method after fluorophore optimization (Figures 2, 3) delivers an optimal performance in terms of specificity which, at least for the TrkA case, is shared from the long versions of these tags, like ACP, to the smaller ones like S6 tag (Supplementary Figure 3).

## Data Availability Statement

The raw data supporting the conclusions of this article will be made available by the authors, without undue reservation, to any qualified researcher.

## Author Contributions

LM and GS conceived the project. RA, DC, GS, and LM designed experiments. RA, DC, MC, LC, FB, CR, GS, and LM performed experiments. RA, DC, SL, GS, and LM analyzed data. RA, DC, SM, SL, AC, CM, LM, and GS discussed data. LM, RA, and GS wrote the manuscript with contributions from all authors.

## Conflict of Interest

The authors declare that the research was conducted in the absence of any commercial or financial relationships that could be construed as a potential conflict of interest.
