# Supplementary material for: Fluorolabeling of the PPTase-Related Chemical Tags: Comparative Study of Different Membrane Receptors and Different Fluorophores in the Labeling Reactions
Source: Front Mol Biosci. 2020 Aug 7;7:195. doi: 10.3389/fmolb.2020.00195 (PMC7426934; doi:10.3389/fmolb.2020.00195)
Supplement: Supplementary file 1 [file Data_Sheet_1.docx]

**Supplementary Material**

**Supplementary Methods**

***Constructs***

S6-fusion constructs of TrkA and p75NTR receptors were described previously [1], [2]; S6-VEGFR2 construct was described recently [3].

***Cell culture***

SH-SY5Y cells (a kind gift from Fondazione EBRI, Rome, Italy) were grown in DMEM/F-12 medium supplemented with 10% Fetal Bovine Serum, 1% Penicillin-Streptomycin, 1% L-Glutamine and 25 mM HEPES. Cells were transfected with plasmids carrying the constructs of interest by using the Lipofectamine^TM^ 2000 reagent (Termo Fisher Scientific), according to the manufacturer’s instructions; or they were transduced using third generation lentiviral particles prepared as described in ref. [1], and carrying the constructs of interest under the control of doxycycline for expression, which was typically achieved with 0.05 μg/ml to 1 μg/ml doxycycline addition in the cell medium [1]. DRG tissues were extracted from wild-type B6129 P3-P4 neonatal mice; the procedure was approved by the Italian Ministry of Health (protocol n. 917) and conducted in compliance with the Italian National Research Council guidelines. For compartmented culture, PDMS microfluidic chambers (RD450 and RD150, Xona Microfluidics) were sealed to glass coverslips coated with 30μg/ml Poly-D-Lysine (Sigma Aldrich) and 5μg/ml laminin (Thermo Fisher). 10 µl of dissected neurons were loaded in the soma compartment at a concentration of 2.5 to 4.5 million cells per ml. Neurons were maintained at 37°C, 5% CO_2_ in Primary Neuron Basal medium (PNBM, Lonza) supplemented with 1% L-glutamine (Lonza), 0.1% Gentamicin Sulfate/Amphotericin-B (Lonza), 2% NSF-1 (Lonza), 2.5 µM AraC (Sigma Aldrich) and 100 ng/ml NGF (Alomone Labs). After 24h from seeding, an NGF gradient (50ng/ml NGF in the soma and 100ng/ml NGF in the axon compartment) was used to drive axon elongation in the microchannels connecting the two compartments. Medium was renewed every day with a pre-warmed fresh one, maintaining the NGF gradient.

***Immunoblotting and Immunoprecipitation***

48 h after transfection, SHSY5Y cells transfected with S6-TrkA-EGFP, S6-P75NTR-EGFP and S6-VEGFR2 constructs were starved for two hours in non-supplemented medium containing 0.5% BSA and labelled for 30 minutes at 37°C with a mix containing 2 µM Sfp synthase, 10 mM MgCl_2_ and 10 µM CoA-biotin in starvation medium. The cell monolayer was scraped in RIPA buffer (Sigma Aldrich) plus protease inhibitors (EDTA-free Protease Inhibitor Cocktail, Sigma Aldrich), and 200-500 µg of cell lysate incubated with 100 µl Dynabeads MyOne Streptavidin C1 (Thermo Fisher Scientific) for 1h at room temperature. The beads were boiled at 100°C for 10 min in Laemmli buffer 2X and loaded on a gel 4-20 % (7.5% for VEGFR2) Criterion TGX Stain-free pre-cast gel, Biorad). This was lately transferred onto a PVDF membrane (Trans-Blot Turbo, Biorad), blocked for 1 h with Tris Buffered Saline + 0.05% Tween-20 (TBST) supplemented with 5% w/v BSA (Sigma), incubated 2h with Streptavidin-HRP conjugate (1:15000 in TBST, GE Healthcare) and washed 3 times with TBST. Blotted membranes were incubated with Clarity Western ECL substrate (Biorad) and signal detected with ChemiDoc MP imaging system (Bio-Rad). Other 25-50 μg of each cell lysate were separated by SDS–PAGE and probed with anti-TrkA (06-574, Millipore, dilution 1:1000), P75NTR antibody (07-476, Millipore, 1:1000) or with anti-VEGFR2 antibody (9698, Cell Signaling 1:1000) in a Western blot.

***Production and purification of fluoNGF***

90 µg of purified NGF-YBBR prepared as previously described [4] were diluted in PBS and spun at 7000 rpm for 1 min at 4 °C. The following reagents were then added: 73 µM CoA-Alexa488 or CoA-Abberior488, 17µM Sfp Synthase, 36 mM MgCl_2_ in PBS up to 270 µl final volume. The reaction was performed in the same tube were NGF was stored. The solution was incubated at 37 °C and 350 rpm for 30 min, the tube was placed in ice before HPLC purification, according to previous procedures [4], [5]. Briefly, we performed a cation exchange HPLC, using a Propac SCX-20 column (Dionex, Thermo Fisher Scientific) with 100 mM HNa_2_PO_4_ mobile phase with 0 to 1 M NaCl gradient. The temperature was kept at 4 °C during run and fraction collection. fluoNGF was separated from the non-reacted neurotrophin and from the free fluorophore by measuring absorbance at 280 nm and at 488 nm, and exploiting the shift in elution time displayed by the reacted neurotrophin due to the negative charge introduced by fluorophore conjugation. Purified fluoNGF was quantified by spectrofluorimetry from comparison of its fluorescence signal to a calibration curve obtained by serial dilutions of CoA-Alexa488 or CoA-Abberior488 of known concentration. Purified fluoNGF was stored in the HPLC elution buffer at 4 °C protected from light.

**Supplementary Tables and figures**

***Supplementary Table 1.*** List of the main properties of the eight organic dyes investigated in our study. ε: extinction coefficient; ^a^ logD value measure of hydrophobicity [6]–[8], calculated on the not hydrolyzed maleimide derivative; ^b^ calculated using the online property calculator service molinspiration ([www.molinspiration.com](http://www.molinspiration.com)); ^c^ structure provided by the supplier.

| Fluorophore | Excitation wavelength (nm) | Emission wavelength (nm) | ε (M^-1^ cm^-1^) | Quantum Yield | Net charge at pH 7.4 | logD value at pH 7.4^a^ | Mass added to the protein up­on con­ju­ga­tion to S6 tag (g/mol) |
| --- | --- | --- | --- | --- | --- | --- | --- |
| Abberior 488 | 501 | 524 | 86000 | 0.89 | **-2** | -1.00^b,c^ | **1133.6** |
| Alexa 488 | 495 | 519 | 73000 | 0.92 | **-3** | -6.75 | **981** |
| Atto 488 | 501 | 523 | 90000 | 0.80 | **-1** | -4.25 | **1405** |
| Alexa 568 | 578 | 603 | 88000 | 0.69 | **-2** | 0.94^b^ | **1130** |
| Atto 550 | 554 | 576 | 120000 | 0.80 | **+1** | 6.41 | **1154** |
| Abberior 635P | 633 | 653 | 75000 | 0.92 | **-1** | 0.20^b^ | **1681** |
| Alexa 647 | 650 | 665 | 270000 | 0.33 | **-3** | -4.26 | **1493** |
| Atto 633 | 629 | 657 | 130000 | 0.64 | **+1** | 0.74^b^ | **1112** |

***Supplementary Table 2.*** List of labelling parameters for different preparations (two for NGF-Abberior488 and five for NGF-Alexa488) of fluoNGF.

| Sample | fluoNGF quantities recovered from labelling of 45 µg NGF-YBBR | Net charge of fluoNGF at pH 7.4 (monomer) | Total MW after fluorophore conjugation (monomer) |
| --- | --- | --- | --- |
| NGF-Abberior 488 | 6.96  2.65 | +2 | 15548 Da |
| NGF-Alexa 488 | 1.79  0.50  2.75  4.42  3.71 | +1 | 15395 Da |

**Supplementary Figure and Video Legends**

***Videos S1 and S2 (related to Supplementary Figure 2).*** Videos of S-Qdot-conjugated ACP-TrkA (S1) and S6-TrkA (S2) receptors in a living SHSY5Y cell previously transfected and labelled as described in the Methods section. Total video time: 10.5 seconds each.

***Supplementary Figure 1.*** Western Blot (WB) comparative analysis of S6-TrkA-eGFP, S6-VEGFR2 and S6-P75-eGFP site-specific biotinylation. Transfected cells (or mock-transfected as control) were starved for 2h and treated with the Biotinilation mix (+Biot mix) or with starvation medium only (-Biot mix). Cells were then lysed and lysates pulled down (PD) with Streptavidin magnetic beads and blotted after purification with Strepavidin-HRP conjugate (panel A). Asterisks in panel A highlight the biotinylated S6-TrkA-eGFP and S6-P75-eGFP bands. The same lysates were loaded on a gel and blotted with α-TrkA (panel B), α-VEGFR2 (panel C) or α-P75 (panel D) antibodies.

**
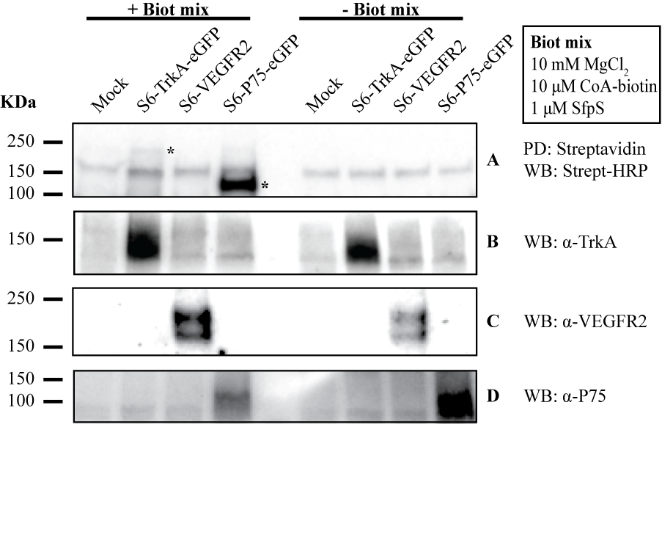
**

***Supplementary Figure 2.* A)** Representative TIRF microscopy image of SHSY5Y cell expressing S6-TrkA labelled with Abberior 635P (the solid white line indicates the cell border, the light blue rectangle indicates the region of background); scale bar: 10 µm. Below: corresponding fluorescence intensity histogram of the background-subtracted cell shown above; the dashed red line indicates the mean value of fluorescence intensity obtained. **B)** Representative TIRF microscopy image of a SHSY5Y cell expressing A1-P75NTR labelled with Abberior 635P (the solid white line indicates the cell border, the light blue rectangle indicates the region of background); scale bar: 10 µm. Below: corresponding fluorescence intensity histogram of the background-subtracted cell shown above; the dashed red line indicates the mean value of fluorescence intensity obtained. Right: Quantification of the mean fluorescence intensity (±s.e.m.) calculated for each fluorophore-conjugated to A1-P75NTR, normalized for its specific brightness value. Differences are not statistically significant within each fluorophore class, following one-way ANOVA test (n=21 cells for Abberior488, n=20 cells for Alexa488, n=28 cells for Atto488, n=9 cells for Atto550, n=16 cells for Alexa568, n=16 cells for Abberior635P, n= 18 for Alexa647, and n=21 cells for Atto633).


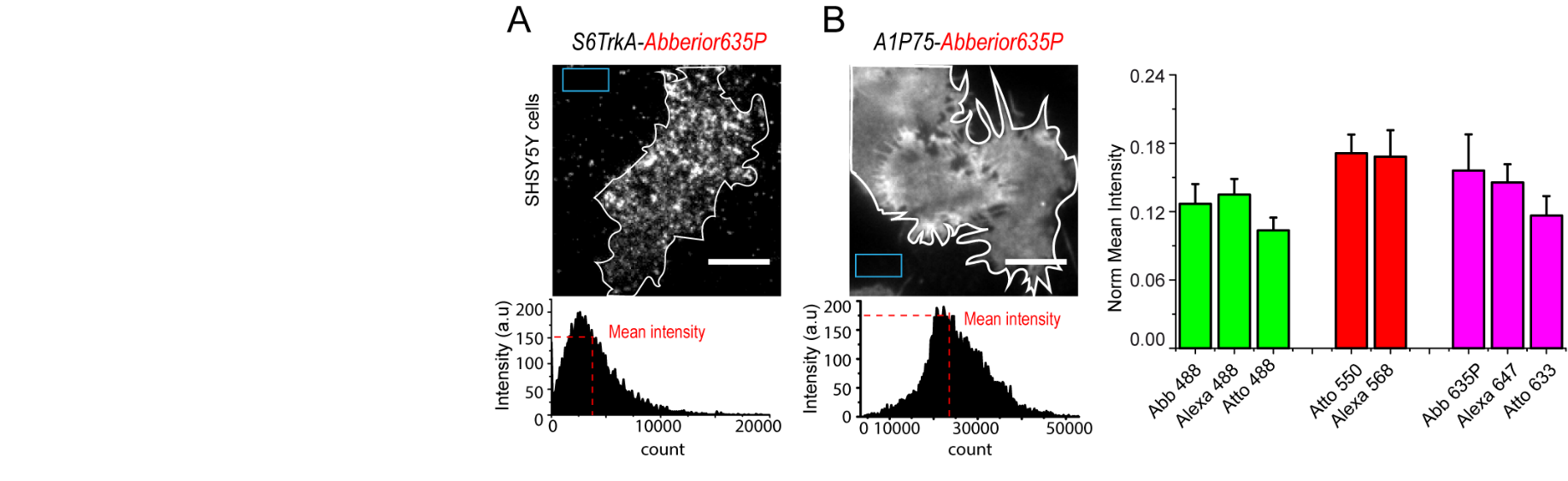


***Supplementary Figure 3.*** On the left, typical TIRF images of Qdot655 and corresponding merge of green epifluorescence (EPI) and DIC images of SHSY5Y cells expressing human S6-tagged TrkA **(A)** and rat ACP-tagged TrkA **(B)** carrying also an EGFP directly fused to the receptor (**A**) or coexpressed by the same plasmid (**B**). The images show the presence of Qdot-labelled receptor spots selectively on EGFP positive cells; scale bar: 10 µm. On the right: quantification of the average receptor spot density, calculated as the number of moving spots at cell surface divided for the cell area, for: **(A)** cells expressing (S6TrkA+) and cells not expressing S6TrkA (S6TrkA-), in absence (S6TrkA+, n=13 cells; S6TrkA-, n=13 cells) and presence (S6TrkA+, n=24 cells; S6TrkA-, n=24 cells) of Sfp Synthase, and **B)** cell expressing ACPTrkA (ACPTrkA+, n=14 cells) and cells not expressing ACPTrkA (ACPTrkA-, n=14 cells). *** P<0.001 according to One-Way Anova test (A) and Mann-Whitney test (B).

**
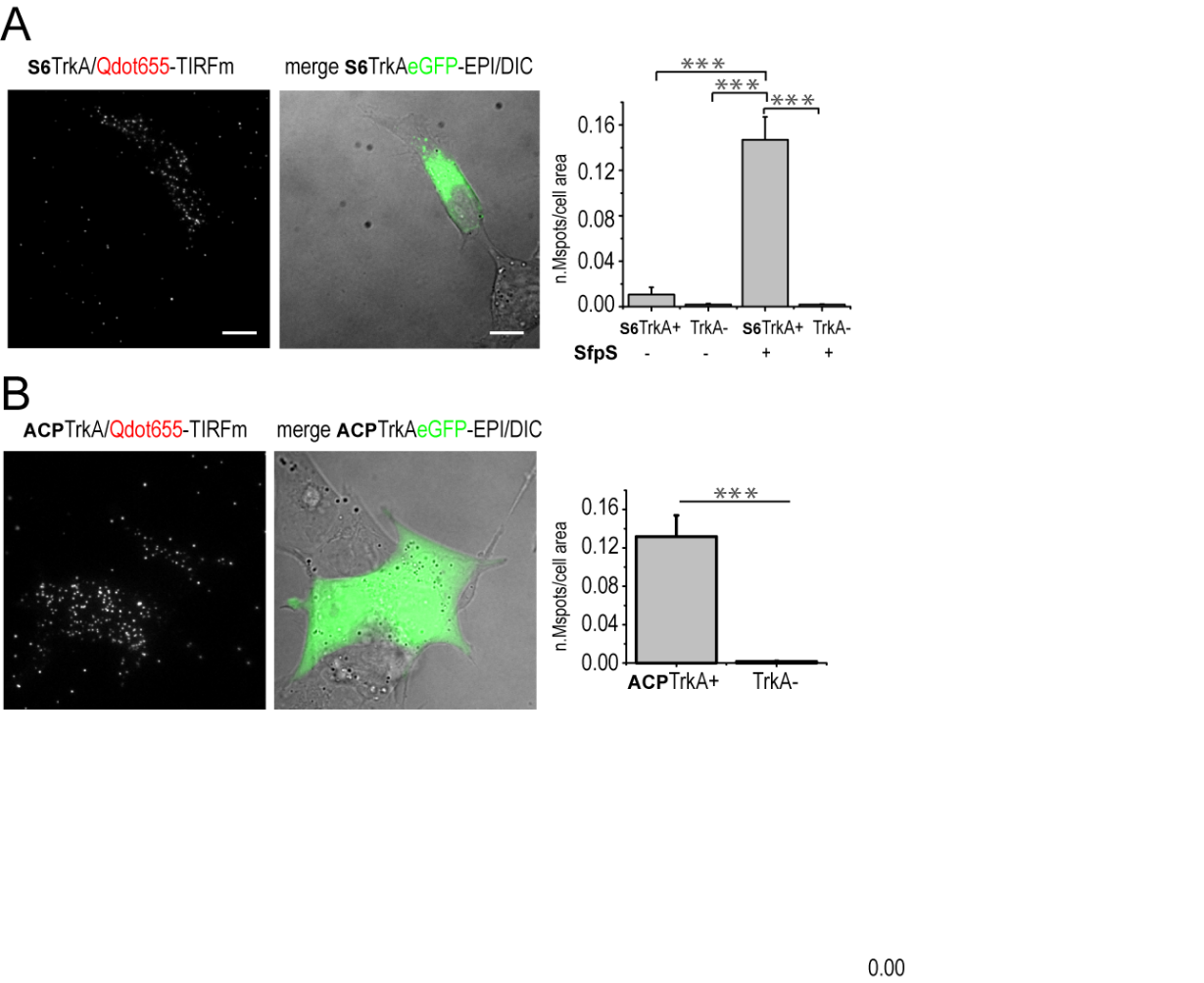
**

**References**

[1] F. Gobbo, F. Bonsignore, R. Amodeo, A. Cattaneo, and L. Marchetti, “Site-specific direct labeling of neurotrophins and their receptors: From biochemistry to advanced imaging applications,” in *Methods in Molecular Biology*, vol. 1727, 2018, pp. 295–314.

[2] L. Marchetti *et al.*, “ Fast-diffusing p75 NTR monomers support apoptosis and growth cone collapse by neurotrophin ligands ,” *Proc. Natl. Acad. Sci.*, 2019.

[3] R. Amodeo *et al.*, “BBA - Molecular Cell Research Molecular insight on the altered membrane trafficking of TrkA kinase dead mutants,” *BBA - Mol. Cell Res.*, vol. 1867, no. 2, p. 118614, 2020.

[4] P. Di Matteo, M. Calvello, S. Luin, L. Marchetti, and A. Cattaneo, “An Optimized Procedure for the Site-Directed Labeling of NGF and proNGF for Imaging Purposes,” *Front. Mol. Biosci.*, vol. 4, no. February, pp. 1–9, 2017.

[5] T. De Nadai *et al.*, “Precursor and mature NGF live tracking: One versus many at a time in the axons,” *Sci. Rep.*, vol. 6, no. February, pp. 1–12, 2016.

[6] L. D. Hughes *et al.*, “Choose Your Label Wisely : Water-Soluble Fluorophores Often Interact with Lipid Bilayers,” vol. 9, no. 2, 2014.

[7] Z. Zhang, D. Yomo, and C. Gradinaru, “Choosing the right fluorophore for single-molecule fluorescence studies in a lipid environment,” *Biochim. Biophys. Acta - Biomembr.*, vol. 1859, no. 7, pp. 1242–1253, 2017.

[8] L. C. Zanetti-Domingues, C. J. Tynan, D. J. Rolfe, D. T. Clarke, and M. Martin-Fernandez, “Hydrophobic Fluorescent Probes Introduce Artifacts into Single Molecule Tracking Experiments Due to Non-Specific Binding,” *PLoS One*, vol. 8, no. 9, 2013.
